# The effect of aircraft noise on sleep disturbance among the residents near a civilian airport: a cross-sectional study

**DOI:** 10.1186/s40557-016-0123-2

**Published:** 2016-09-02

**Authors:** Kyeong Min Kwak, Young-Su Ju, Young-Jun Kwon, Yun Kyung Chung, Bong Kyu Kim, Hyunjoo Kim, Kanwoo Youn

**Affiliations:** 1 Department of Occupational and Environmental Medicine, Hallym University Sacred Heart Hospital, Anyang, South Korea; 2 Department of Occupational and Environmental Health, Graduate School of Public Health, Seoul National University, Seoul, South Korea; 3 Department of Occupational and Environmental Medicine, Ewha Womans University Mokdong Hospital, Seoul, South Korea; 4 Department of Occupational and Environmental Medicine, Wonjin Institute for Occupational and Environmental Health Green Hospital, Seoul, South Korea

**Keywords:** Aircraft, Noise, Sleep disturbance, Insomnia, Daytime hypersomnia

## Abstract

**Background:**

Aircraft noise is a major environmental noise problem. This study was conducted in order to investigate the relationship between sleep disturbance and exposure to aircraft noise on the residents who are living near an airport.

**Methods:**

There were 3308 residents (1403 in the high exposure group, 1428 in the low exposure group, and 477 in the non-exposure group) selected as the subjects for this study. The Insomnia severity Index (ISI) and Epworth Sleepiness Scale (ESS) questionnaires were used to evaluate sleep disturbance.

**Results:**

The mean ISI and ESS scores were 6.9 ± 6.4 and 5.5 ± 3.7, respectively, and the average scores were significantly greater in the aircraft noise exposure group, as compared to the non-exposure group. The percentage of the abnormal subjects, which were classified according to the results of the ISI and ESS, was also significantly greater in the noise exposure group, as compared to the control group. The odd ratios for insomnia and daytime hypersomnia were approximately 3 times higher in the noise exposure group, as compared to the control group.

**Conclusions:**

The prevalence of insomnia and daytime hypersomnia was higher in the aircraft noise exposure group, as compared to the control group. Further study is deemed necessary in order to clarify the causal relationship.

## Background

Noise is defined as any unwanted, or mentally or physically harmful sound [1]. As described in its definition, noise involves psychological factors as well as physiological features. As a result, it may unfavorably affect a person’s hearing ability or cause various health problems, such as hypertension [2], myocardial infarction [3], psychological disease [4], and sleep disturbance [5].

With the rapid growth of air traffic, aircraft noise has recently become a major environmental noise problem. The aircraft noise can affect a person’s hearing ability [6], blood pressure [7], mental health [8], and sleep quality [9, 10]. A continuous exposure to aircraft noise increases the frequency of waking up during sleep and decreases slow-wave sleep, sometimes called deep sleep. This condition can cause a decreased quality of sleep and sleep disturbance [9]. Sleep disturbance is an important health issue and it has been associated with other health problems [10]. Sleep deprivation, which is caused by sleep disturbance, is related to obesity, hypertension, diabetes, cardiovascular disease, depression, and increased risk of mortality [11–15]. Many studies have been conducted on the effect of aircraft noise on sleep [16, 17]; however, the population sizes of most studies are insufficient. There are only a few studies conducted in the large populations of more than 1000 subjects [18, 19]. Large population studies that directly evaluate sleep disturbance have not sufficiently supported the clear correlation between noises and sleep disturbance.

This study conducted a survey on more than 3000 subjects by using a structured questionnaire. The purpose of this study is to investigate the relationship between sleep disturbance and exposure to aircraft noise on residents who are living near an airport.

## Methods

### Noise measurement

This study did not measure the aircraft noise level directly, but instead, we used the result of the aircraft noise measurement in the official announcement of the Seoul Regional Aviation Administration (SRAA) [20] that was issued on October 8, 2010. This announcement was based on the noise measurement of the areas near the Gimpo International Airport that was performed by noise specialists in 2008. For this measurement, 50 sites were chosen to measure the aircraft noise, and the Weighted Equivalent Continuous Perceived Noise Level (WECPNL) was used as the noise metric. The WECPNL was recommended by the International Civil Aviation Organization (ICAO) for measuring the aircraft noise [21]. The WECPNL used in Korea is defined as follows [22]:

$$ \mathrm{WECPNL} = \kern0.5em {\overline{\mathrm{L}}}_{\mathrm{A}}\kern0.5em +\kern0.5em 10 \log \kern0.5em \left({\mathrm{N}}_2\kern0.5em +\kern0.5em 3{\mathrm{N}}_3\kern0.5em +\kern0.5em 10\left({\mathrm{N}}_1\kern0.5em +\kern0.5em {\mathrm{N}}_4\right)\right) - 27, $$

where ${\overline{\mathrm{L}}}_{\mathrm{A}}$ is the energy mean of all maximum aircraft noise level during daytime. N_1_ is the number of flight events during midnight from 00:00 to 07:00, N_2_ is the number of events during daytime from 07:00 to 19:00, N_3_ is the number of flight events during nighttime from 19:00 to 22:00, and N_4_ is the number of flight events during late nighttime from 22:00 to 24:00.

### Study subjects

This study has chosen the aircraft noise exposure areas based on the official announcement of SRAA. This announcement divided the areas near the Gimpo International Airport into 3 districts (type 1 [95+ WECPNL]), type 2 [90–95 WECPNL], and type 3 [75–90 WECPNL]) based on the aircraft noise level. There were no residents living in type 1 and 2 districts. The type 3 district was divided again into 3 subdistricts (‘Ga’ [85–90 WECPNL], ‘Na’ [80–85 WECPNL], and ‘Da’ [75–80 WECPNL]).

**Fig. 1:**
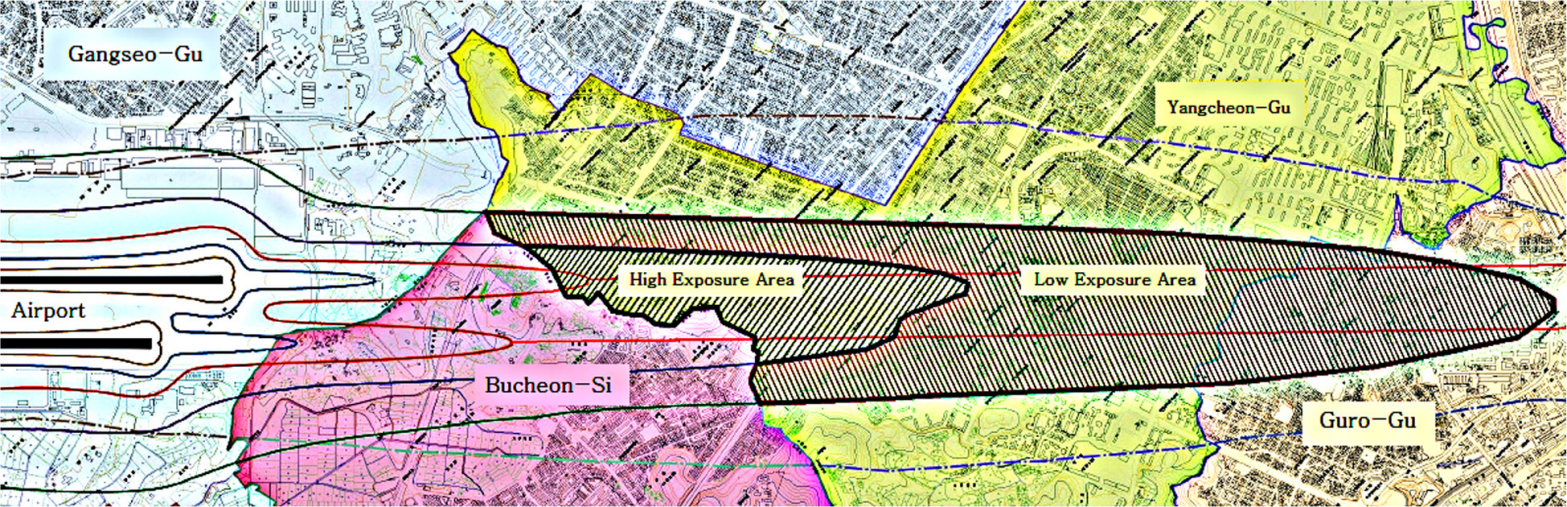
Aircraft noise map in the vicinity of Gimpo International Airport

According to this official announcement by SRAA, the areas in Seoul City near the Gimpo International Airport, which required measurement for noise monitoring, were selected for this study. This study classified ‘Ga’ and ‘Na’ into a high noise exposure group (80-90 WECPNL) and ‘Da’ into a low noise exposure group (75-80 WECPNL) (Fig. 1). ‘A’-dong was selected as the control area with similar demographic, socioeconomic, and geologic characteristics, and without aircraft noise, as it is far from the airport. However, the control area did not have a noise measurement result.

This study was conducted as a door-to-door visit by the researchers from March to April 2015 in order to investigate the effect of aircraft noise on the health of the residents living near the Gimpo International Airport. Adults, who are 20 years old and above, were included in the study, but those who are older than 75 years old were excluded. A total of 3531 residents (1516 in the high exposure group, 1515 in the low exposure group, and 500 in the non-exposure group) participated in this survey. The 166 residents (61 in the high exposure group, 90 in the low exposure group, and 15 in the non-exposure group), who had been treated for depression within 1 year, were excluded from the study. In addition, 57 residents (27 in the high exposure group, 22 in the low exposure group, and 8 in the non-exposure group), whose questionnaire missed a significant amount of information, were also excluded from the study. Finally, 3308 residents (1428 in the high exposure group, 1403 in the low exposure group, and 477 in the non-exposure group) were selected as subjects for the analysis.

### Survey tool

Survey tools for insomnia and daytime hypersomnia were used to evaluate sleep disturbance. The Insomnia Severity Index (ISI) [23, 24] was used to measure insomnia. The ISI is a self-reported questionnaire that consists of 7 questions for evaluating the difficulties of sleep onset and sleep maintenance, satisfaction with current sleep pattern, interference with daily functioning, noticeability of impairment attributed to the sleep problem, and degree of distress or concern caused by the sleep problem. Each question is scored between 0 and 4, and a higher score means a more severe status. The total score is ranged between 0 and 28. A score of 0-7 is considered as normal, 8-14 is considered as sub-threshold insomnia, 15–21 is considered as moderate insomnia, and 22–28 is considered as severe insomnia. The Epworth Sleep Scale (ESS) [25] was used in order to measure daytime hypersomnia. ESS uses a scoring system from 0 to 3 to indicate the degree of drowsiness in 8 different situations. A score of 3 indicates that a person feels sleepy the most. The total score is ranged from 0 to 24 and a score above 10 is considered as daytime hypersomnia.

### Analysis method

A technical analysis was performed in order to investigate the demographic and sociological characteristics, as well as the degree of sleep disturbance of the subjects. ANOVA and Chi-square test were used to investigate if there was any difference in the demographic and sociological characteristics between the groups. The Mantel-Haenszel Chi-square test was performed to investigate if the demographic and sociological characteristics, as well as the degree of noise exposure, were related to insomnia or daytime hypersomnia. In addition, the results that showed a significance in the univariate analysis (age, sex, education level, residency period, smoking, drinking, exercise, and medical history) were corrected by using a multiple logistic regression model. The odds ratio and 95 % confidence interval were obtained for the effect of the exposure degree on insomnia and daytime hypersomnia.

## Results

### General characteristics of the subjects

There were 3308 subjects, and their characteristics were analyzed by using a frequency analysis. The female subjects accounted for 66.4 % among the entire subjects, which were twice the number of the male subjects. The mean age of the subjects was 50.5 years old. Based on the age groups, 764 (23.1 %) subjects aged 60–69 years old accounted for the majority of the subjects, closely followed by the group with 739 (22.3 %) subjects aged 50–59 years old. For the education level, high school drop-out or graduate took up the greatest portion with a total of 1407 subjects (42.5 %). For the residency period, the greatest number of subjects, which was 826 (28.7 %), had lived for over 15 years in their residences.

A total of 1253 (37.9 %) subjects answered that they drink, while 489 (14.8 %) subjects answered that they are current smokers. A great number of subjects (1515, 45.8 %) answered that they exercise regularly. There were 283 (8.6 %) subjects who had been hospitalized or had undergone operations in the previous year.

### Comparison of general characteristics by noise exposure groups

For sex, the male subjects accounted for a significantly greater portion in the high exposure group (36.1 %) than the low exposure group (31.4 %) and the control group (32.1 %). The mean age and age distribution did not show any significant difference between the groups.

The education level results showed that the subjects, who received a high school education level or an even higher education, were smaller in numbers in the high exposure group and low exposure group, as compared to the control group (69.1 % vs 71.3 % vs 81.4 %), and the difference was statistically significant. For the residency period, 29.0 % of the subjects in the high exposure group and 31.4 % in the low exposure group lived in the area for 15 years or longer, which was significantly higher than that of the control group (20.3 %). For the drinking factor, 39.4 % of the subjects in the high exposure group and 40.5 % in the control group answered that they drink, which was significantly higher than that of the low exposure group (35.4 %). There was no significant difference in the results for the smoking factor between the groups. The 45.6 % of the subjects in the high exposure group and 44.3 % of the subjects in the low exposure group answered that they exercise regularly, which was significantly lower than that of the control group (50.9 %). The 10.1 % of the subjects in the high exposure group had been hospitalized or had undergone operations in the previous year, which was significantly higher than that of the low exposure group (7.6 %) and the control group (6.5 %).

### Comparison of ISI and ESS results by the noise exposure groups

The mean score of the ISI in all subjects was 6.9 ± 6.4. There were 1956 (59.1 %) subjects in the normal group, 897 (27.1 %) subjects in the sub-threshold insomnia group, 382 (11.6 %) subjects in the moderate insomnia group, and 73 (2.2 %) subjects in the severe insomnia group. The mean score of ESS was 5.5 ± 3.7. There were 2853 (86.2 %) subjects in the normal group, and 455 (13.8 %) subjects in the daytime hypersomnia group.

The ISI scores of the three groups were compared, and the results showed that the mean score increased from the control group to the high exposure group, thereby showing 4.1 ± 5.1 in the control group, 7.2 ± 6.5 in the low exposure group, and 7.6 ± 6.4 in the high exposure group. The post-hoc results showed that the difference of the scores between the control group and low exposure group, and between the control group and high exposure group were statistically significant. The percentage of the subjects with moderate or severe insomnia increased from the control group to high exposure group, thereby showing 26 (5.4 %) for the control group, 195 (13.9 %) for the low exposure group, and 234 (16.4 %) for the high exposure group. The Mantel-Haenszel Chi-square test results showed that the percentage of the subjects with insomnia had a statistically significant difference among the groups.

Likewise, the ESS scores of the three groups were compared, and the results showed that the mean score also increased from the control group to the high exposure group, thereby showing 4.1 ± 3.0 in the control group, 5.4 ± 3.7 in the low exposure group, and 6.0 ± 3.8 in the high exposure group. The post-hoc analysis results showed that the difference between all groups were statistically significant. The percentage of the subjects with daytime hypersomnia increased from the control group to high exposure group, thereby showing 26 (5.5 %) for the control group, 189 (13.5 %) for the low exposure group, and 240 (16.8 %) for the high exposure group. The Mantel-Haenszel Chi-square test results showed that the percentage of the subjects with daytime hypersomnia had a statistically significant difference among the groups (Table 1).

**Table 1:** General characteristics and Insomnia Severity Index (ISI)/Epworth Sleepiness Scale (ESS) results by noise exposure groups

Characteristics		All groups	Control	Low-exposure (75-80 WECPNL)	High-exposure (80-90 WECPNL)	*p*-value
		*n* = 3308(%)	*n* = 477(%)	*n* = 1403(%)	*N* = 1428(%)	
Sex^a)^	Male	1111(33.6)	153(32.1)	443(31.6)	515(36.1)	0.0308
	Female	2197(66.4)	324(67.9)	960(68.4)	913(63.9)	
Mean age(years)^b)^		50.5 ± 14.2	50.5 ± 14.4	50.6 ± 14.1	50.4 ± 14.2	0.9116
Age(years)^a)^	20-29	268(8.1)	44(9.2)	110(7.8)	114(8.0)	0.4197
	30-39	585(17.7)	81(17.0)	240(17.1)	264(18.5)	
	40-49	656(19.8)	77(16.1)	300(21.4)	279(19.5)	
	50-59	739(22.3)	121(25.4)	297(21.2)	321(22.5)	
	60-69	764(23.1)	108(22.6)	330(23.5)	326(22.8)	
	70-74	296(9.0)	46(9.6)	126(9.0)	124(8.7)	
Education^a)^	Never	77(2.3)	4(0.8)	26(1.8)	47(3.3)	<0.0001
	Elementary school	343(10.4)	28(5.9)	134(9.6)	181(12.7)	
	Middle school	512(15.5)	57(11.9)	242(17.3)	213(14.9)	
	High school	1407(42.5)	225(47.2)	552(39.3)	630(44.1)	
	College or more	969(29.3)	163(34.2)	449(32.0)	357(25.0)	
Residency period(year)^a)^	≥15	826(28.7)	86(20.3)	382(31.4)	358(29.0)	<0.0001
	10-14	655(22.8)	120(28.4)	287(23.6)	248(20.1)	
	5-9	679(23.6)	101(23.9)	260(21.4)	318(25.7)	
	<5	716(24.9)	116(27.4)	288(23.7)	312(25.2)	
Drinking^a)^	No	2055(62.1)	284(59.5)	906(64.6)	865(60.6)	0.0408
	Yes	1253(37.9)	193(40.5)	497(35.4)	563(39.4)	
Smoking^a)^	Never	2546(77.4)	369(77.4)	1102(78.5)	1075(75.3)	0.2182
	Past smoker	273(8.2)	43(9.0)	101(7.2)	129(9.0)	
	Current smoker	489(14.8)	65(13.6)	200(14.3)	224(15.7)	
Regular Exercise^a)^	No	1793(54.2)	234(49.1)	782(55.7)	777(54.4)	0.0398
	Yes	1515(45.8)	243(50.9)	621(44.3)	651(45.6)	
Operation or hospitalization within 1 year^a)^	No	3025(91.4)	446(93.5)	1296(92.4)	1283(89.9)	0.0123
	Yes	283(8.6)	31(6.5)	107(7.6)	145(10.1)	
ISI^c)^	Mean^b)^	6.9 ± 6.4	4.1 ± 5.1	7.2 ± 6.5	7.6 ± 6.4	<0.0001
	Normal	1956(59.1)	376(78.8)	782(55.7)	798(55.9)	<0.0001
	Sub-threshold insomnia	897(27.1)	75(15.7)	426(30.4)	396(27.7)	
	Moderate insomnia	382(11.6)	25(5.2)	155(11.1)	202(14.2)	
	Severe insomnia	73(2.2)	1(0.2)	40(2.8)	32(2.2)	
ESS^c)^	Mean^b)^	5.5 ± 3.7	4.1 ± 3.0	5.4 ± 3.7	6.0 ± 3.8	<0.0001
	Normal	2853(86.2)	451(94.5)	1214(86.5)	1188(83.2)	<0.0001
	Daytime hypersomnia	455(13.8)	26(5.5)	189(13.5)	240(16.8)	

^a)^By Chi-square test

^b)^By ANOVA

^c)^By Mantel-Haenszel Chi-square test

### Comparison of ISI and ESS results by the characteristics of the subjects

The percentages of the subjects, who were considered as having sub-threshold insomnia, moderate insomnia, and severe insomnia based on ISI, were significantly higher in females (28.7 %, 12.6 %, and 2.8 %, respectively) than in males. The subjects with more severe insomnia showed greater mean age. Likewise, the percentages of the older subjects, who were considered as having sub-threshold insomnia, moderate insomnia, and severe insomnia, were higher than the younger subjects. The lower education level was associated with a high percentage of the subjects with insomnia, thereby disregarding the subjects with no education. The subjects, who have lived longer in the area, showed more insomnia. Meanwhile, the subjects, who had been hospitalized or had undergone operations in the previous year, had more insomnia. The subjects who are non-smokers and non-drinkers, as well as the subjects who exercise regularly, had more insomnia.

Based on the ESS, the percentage of the subjects suffering from daytime hypersomnia was 14.4 % in females, which was significantly higher than in males. The subjects with daytime hypersomnia showed greater mean age. The older subjects also showed more daytime hypersomnia. The lower education level was associated with a high percentage of subjects with daytime hypersomnia. The subjects, who have lived longer in the area, showed more daytime hypersomnia. Meanwhile, the subjects, who had been hospitalized or had undergone an operation in the previous year, showed more insomnia. No statistically significant relationship between smoking, drinking, exercise, and daytime hypersomnia was confirmed (Table 2).

**Table 2:** Insomnia Severity Index (ISI)/Epworth Sleepiness Scale (ESS) according to subject characteristics

Characteristics		ISI				ESS	
		Normal	Sub-threshold insomnia	Moderated insomnia	Severe insomnia	Normal	Daytime hypersomnia
Sex	Male	726(65.4)	267(24.0)	106(9.5)	12(1.1)^c)^***	972(87.5)	139(12.5)^a)^
	Female	1230(56.0)	630(28.7)	276(12.6)	61(2.8)	1881(85.6)	316(14.4)
Mean age(years)^b)^		48.7 ± 14.3	52.0 ± 13.6	54.6 ± 13.2	57.7 ± 11.9^**^	49.9 ± 14.2	54.2 ± 13.1^**^
Age(years)^c)^	20-29	200(74.6)	56(20.9)	11(4.1)	1(0.4)****	250(93.3)	18(6.7)****
	30-39	389(66.5)	140(23.9)	52(8.9)	4(0.7)	527(90.1)	58(9.9)
	40-49	409(62.3)	164(25.0)	70(10.7)	13(2.0)	570(86.9)	86(13.1)
	50-59	420(56.8)	218(29.5)	85(11.5)	16(2.2)	631(85.4)	108(14.6)
	60-69	394(51.6)	229(30.0)	112(14.7)	29(3.8)	637(83.4)	127(16.6)
	70-74	144(48.7)	90(30.4)	52(17.6)	10(3.4)	238(80.4)	58(19.6)
Education^c)^	Never	41(53.2)	22(28.6)	12(15.6)	2(2.6)****	63(81.8)	14(18.2)****
	Elementary School	174(50.7)	104(30.3)	58(16.9)	7(2.1)	279(81.3)	64(18.7)
	Middle school	267(52.1)	150(29.3)	74(14.5)	21(4.1)	434(84.8)	78(15.2)
	High school	849(60.3)	365(25.9)	160(11.4)	33(2.4)	1199(85.2)	208(14.8)
	College or more	625(64.5)	256(26.4)	78(8.1)	10(1.0)	878(90.6)	91(9.4)
Residency period(year)^c)^	≥15	446(54.0)	253(30.6)	108(13.1)	19(2.3)****	685(82.9)	141(17.1)***
	10-14	381(58.2)	179(27.3)	76(11.6)	19(2.9)	571(87.2)	84(12.8)
	5-9	418(61.6)	165(24.3)	80(11.8)	16(2.4)	592(87.2)	87(12.8)
	<5	442(61.7)	195(27.2)	68(9.5)	11(1.5)	625(87.3)	91(12.7)
Drinking	No	1164(56.6)	573(27.9)	265(12.9)	53(2.6)^c)^****	1776(86.4)	279(13.6)^a)^
	Yes	792(63.2)	324(25.9)	117(9.3)	20(1.6)	1077(86.0)	176(14.0)
Smoking^c)^	Never	1474(57.9)	708(27.8)	300(11.8)	64(2.5)***	2190(86.0)	356(14.0)
	Past smoker	180(65.9)	68(24.9)	24(8.8)	1(0.4)	229(83.9)	44(16.1)
	Current smoker	302(61.8)	121(24.7)	58(11.9)	8(1.6)	434(88.8)	55(11.2)
Regular Exercise	No	1111(62.0)	454(25.3)	199(11.1)	29(1.6)^c)^****	1532(85.4)	261(14.6)^a)^
	Yes	845(55.8)	443(29.2)	183(12.1)	44(2.9)	1321(87.2)	194(12.8)
Operation or hospitalization within 1 year	No	1830(60.5)	801(26.5)	329(10.9)	65(2.2)^c)^****	2625(86.8)	400(13.2)^a)^*
	Yes	126(44.5)	96(33.9)	53(18.7)	8(2.8)	228(80.6)	55(19.4)
Noise exposure Group^c)^	Control	376(78.8)	75(15.7)	25(5.2)	1(0.2)****	451(94.6)	26(5.4)****
	Low-exposure	782(55.7)	426(30.4)	155(11.1)	40(2.8)	1214(86.5)	189(13.5)
	High-exposure	798(55.9)	396(27.7)	202(14.2)	32(2.2)	1188(83.2)	240(16.8)

^a)^By Chi-square test

^b)^By ANOVA

^c)^By Mantel-Haeszel Chi-square test

*p < 0.05, **p < 0.001, ***p for trend < 0.05, ****p for trend < 0.001

### Multiple logistic regression for insomnia and daytime hypersomnia

The variables that showed significance in the univariate analysis were corrected by using the multiple logistic regression model. The odds ratio and 95 % confidence interval for the degree of noise exposure and sleep disturbance were obtained. For insomnia, the variables, including sex, age, education level, and residency period, were corrected in the first regression model. The other variables, including operation and hospitalization history for the previous year, smoking, drinking, and regular exercise performance, were additionally corrected in the second model.

The risk of insomnia was 3.45 times (95 % CI 2.64-4.50) higher in the low exposure group and 3.24 times (95 % CI 2.48-4.22) higher in the high exposure group, as compared to that of the control group. The risk of insomnia was 3.41 times (95 % CI 2.61-4.46) higher in the low exposure group and 3.26 times (95 % CI 2.50-4.25) in the high exposure group after additionally correcting the factors of operation and hospitalization history, smoking, drinking, and regular exercise (Model 2), as compared to that of the control group. The female subjects showed a significantly greater risk of insomnia than the males in both Model 1 (OR 1.51, 95 % CI 1.30-1.77) and Model 2 (OR 1.55, 95 % CI 1.24-1.94). The older aged group had a greater risk of insomnia than the younger aged group, and the odds ratio increased with age. However, the risk of insomnia was not significantly different according to the education level and residency period in both Models 1 and 2. The risk of insomnia was 1.71 times (95 % CI 1.35-2.17) greater in the subjects, who had been hospitalized or had undergone operations in the previous year, than the subjects who had not. For the lifestyle habits, the risk of insomnia was not significantly different according to smoking or drinking factors. However, the subjects who regularly exercised had 1.3 times (1.12-1.50) greater risk of insomnia than those who do not (Table 3).

**Table 3:** Multiple logistic regression model for Insomnia Severity Index (ISI) according to subject characteristics

Characteristics		Model 1		Model 2	
		OR	95 % CI	OR	95 % CI
Sex	Male	1.0		1.0	
	Female	1.54	1.31-1.81	1.57	1.24-1.97
Age(years)	20-29	1.0		1.0	
	30-39	1.39	0.95-2.04	1.40	0.95-2.06
	40-49	1.74	1.22-2.50	1.73	1.21-2.49
	50-59	2.27	1.59-3.25	2.17	1.51-3.12
	60-69	2.97	2.05-4.30	2.76	1.89-4.02
	70-74	3.88	2.52-5.98	3.64	2.35-5.65
Education	Never	1.0		1.0	
	Elementary school	1.07	0.64-1.77	1.09	0.65-1.81
	Middle school	1.22	0.74-2.01	1.25	0.76-2.07
	High school	1.15	0.70-1.89	1.19	0.73-1.96
	College or more	1.17	0.69-1.96	1.18	0.73-1.98
Residency period(year)	<5	1.0		1.0	
	5-9	0.93	0.75-1.16	0.93	0.75-1.16
	10-14	1.02	0.82-1.27	1.01	0.81-1.26
	≥15	0.96	0.77-1.19	0.96	0.77-1.19
Noise exposure group	Control	1.0		1.0	
	Low-exposure	3.45	2.64-4.50	3.41	2.61-4.46
	High-exposure	3.24	2.48-4.22	3.26	2.50-4.25
Drinking	No			1.0	
	Yes			0.98	0.82-1.16
Smoking	Never			1.0	
	Past smoker			0.86	0.62-1.21
	Current smoker			1.16	0.88-1.53
Regular Exercise	No			1.0	
	Yes			1.25	1.07-1.45
Operation or hospitalization within 1 year	No			1.0	
	Yes			1.75	1.37-2.25

For daytime hypersomnia, the variables that showed significance in the univariate analysis were also corrected by using a multiple logistic regression model. The variables, including sex, age, education level, and residency period, were corrected in the first regression model. Another variable of operation and hospitalization history for the previous year was additionally corrected in the second model. The results showed a similar pattern as those in the multivariate analysis of insomnia. The risk of daytime hypersomnia was 2.58 times greater (95 % 1.65-4.04) in the low exposure group and 3.43 times greater (95 % CI 2.20-5.34) in the high exposure group, as compared to the control group. In Model 2, the risk of daytime hypersomnia was still greater in the low and high exposure groups, 2.57 times (95 % CI 1.64-4.03) and 3.39 times (95 % CI 2.17-5.28), respectively, as compared to the control group even after the additional variable of the operation and hospitalization history in the previous year has been corrected. The odds ratio of Model 2 was similar to that of Model 1. The female subjects showed a greater risk of daytime hypersomnia than the males in both Model 1 (OR 1.30, 95 % CI 1.03-1.63) and Model 2 (OR 1.29, 95 % CI 1.03-1.62). The older subjects had a greater risk of daytime hypersomnia, as shown in the results for insomnia. The odds ratio increased with age. The risk of daytime hypersomnia was 1.41 times greater (95 % CI 1.02-1.93) in the subjects, who had been hospitalized or had undergone operations in the previous year, than those who had not (Table 4).

**Table 4:** Multiple logistic regression model for Epworth Sleepiness Scale (ESS) according to subject characteristics

Characteristics		Model 1		Model 2	
		OR	95 % CI	OR	95 % CI
Sex	Male	1.0		1.0	
	Female	1.22	0.97-1.55	1.22	0.97-1.55
Age(years)	20-29	1.0		1.0	
	30-39	2.22	1.07-4.58	2.20	1.06-4.54
	40-49	3.04	1.53-6.03	3.03	1.53-6.01
	50-59	3.25	1.65-6.41	3.19	1.62-6.30
	60-69	3.77	1.89-7.53	3.65	1.83-7.29
	70-74	4.53	2.13-9.62	4.39	2.06-9.33
Education	Never	1.0		1.0	
	Elementary school	1.02	0.52-1.99	1.04	0.53-2.05
	Middle school	0.90	0.46-1.77	0.93	0.47-1.82
	High school	1.16	0.60-2.24	1.20	0.62-2.32
	College or more	0.73	0.36-1.50	0.75	0.37-1.54
Residency period(year)	<5	1.0		1.0	
	5-9	0.88	0.64-1.22	0.89	0.64-1.22
	10-14	0.86	0.63-1.19	0.86	0.62-1.20
	≥15	1.05	0.77-1.43	1.05	0.77-1.43
Noise exposure group	Control	1.0		1.0	
	Low-exposure	2.58	1.65-4.04	2.57	1.64-4.03
	High-exposure	3.43	2.20-5.34	3.39	2.17-5.28
Operation or hospitalization within 1 year	No			1.0	
	Yes			1.41	1.00-1.97

## Discussion

The subjects within the exposed area showed a significantly higher mean of ISI than the subjects within the non-exposed area. The ESS mean also showed significantly higher results in the subjects within the exposed area than the subjects within the non-exposed area. The percentage of insomnia and daytime hypersomnia, which were classified according to the results of the ISI and ESS, was also significantly greater in the subjects within the exposed area than the subjects within the non-exposed area. The multiple logistic regression model reflecting the corrected variables, including sex, age, education level, residency period, lifestyle habits, operation, and hospitalization history, showed approximately 3 times higher risk of insomnia and daytime hypersomnia in the subjects within the exposed area than the subjects within the non-exposed area. In summary, the degree of noise exposure and sleep disturbance showed significant association based on the results.

The number of aircraft arrivals and departures by time from Gimpo International Airport can be found from the Airport Statistics [26] that was published by the Korea Airports Corporation. The average number of flight events daily was 51.6 in the evening from 18:00 to 22:00, and 19.5 after 22:00 during this study period between March and April of 2015. The air services during the evening and nighttime change the depth of sleep, maintain wakefulness, and disturb the process of falling into sleep [27]. This study used WECPNL as the noise metric. The WECPNL is an appropriate metric for reflecting the impact on sleep because the flight events during the evening and nighttime are weighted in this metric. As a result, it can be assumed that the air traffic has a direct impact on the sleep pattern of the residents in the area, where the survey was performed, thereby increasing the risk of sleep disturbance (Table 5).

**Table 5:** Daily average number of flight events in Gimpo International Airport (2015. 3. ~ 2015. 4.)

Time	Daily average number of flight events		
	Arrival	Departure	Total
0:00-6:00	0	0	0
6:00-7:00	0	6.0	6.0
07:00-12:00	46.7	68.0	114.7
12:00-18:00	77.1	78.0	155.1
18:00-22:00	51.6	43.2	94.8
22:00-23:00	19.3	0.1	19.4
23:00-24:00	0.1	0	0.1
Total	194.8	195.3	390.1

The previous studies have confirmed that continuous exposure to noise can increase the risk of sleep disturbance [28–31]. There are a few studies that evaluated the relationship between aircraft noise and sleep disturbance, including a community-based cross-sectional study, which is similar to this study, that was conducted by Kim et al. [9]. The sleep quality of the residents adjacent to the airport was evaluated by using the Pittsburgh Sleep Quality Index (PSQI) [32]. The results showed that the quality of sleep was poor in the residents, who were exposed to the aircraft noise, and there was a greater risk of sleep disturbance.

Sleep is also influenced by the sex and age of a person [33]. In this study, female and older subjects showed significant results in terms of their association with sleep disturbance. The prevalence of sleep disturbance showed a difference according to the education level and residency period of the subjects in the univariate analysis. However, the multiple logistic regression model results did not show a statistical significance after the adjustment of such variables.

The subjects, who had been hospitalized or had undergone operations in the previous year, also showed a higher prevalence of sleep disturbance. The chronic comorbidities and health status that may affect the sleep quality [34] and the reverse effect of sleep disturbance can also be considered. Patients with sleep disturbance are more likely to develop affective disorders [35, 36]. Likewise, the prevalence of the hospitalizations or operations was greater in the noise exposure group than that in the control group. It could be considered as a health effect of the aircraft noise [6–10].

For the lifestyle habits, there was no variable that showed a significant association with the occurrence of daytime hypersomnia. In the univariate analysis, the prevalence of insomnia showed some difference based on the lifestyle habits, but only regular exercise performance showed a significance in the multivariate analysis. The subjects, who exercised regularly, showed a higher prevalence of insomnia, which was different from the general understanding that regular exercise improves the quality of sleep [37, 38]. However, exercise near bedtime changes the circardian phase [39], increases the core body temperature [40], and increases the physiological arousal [41], which would disturb sleep. However, this study did not collect the information on the exercise time, so the relationship could not be confirmed. On the contrary, this is a cross-sectional study and a reverse causation can be suspected. It is possible that people, who experience sleep disturbance, tend to exercise more than others.

There are some limitations to this study. First, the subjects of the exposure group were selected based on the official announcement of the Seoul Regional Aviation Administration 5 years earlier without using a direct noise measurement. As it used the past noise level, the current exposure to the noise could not be accurately reflected, and the possibility of a misclassification could not be ruled out. Second, a subjective method was used to evaluate sleep disturbance rather than objective methods, such as EEG and polysomnography. There was a study that evaluated sleep disturbance by using EEG and polysomnography [42], but these objective methods are practically difficult to use in a large-scale epidemiological study. Third, other factors that might have an impact on sleep, such as drinking coffee and watching television at night, were not taken into consideration.

Despite such limitations, this was a large-scale epidemiological study that enrolled more than 3000 subjects. It was the largest scale study among those on aircraft noise conducted in South Korea. This study was significant, as it was conducted on the residents, who live in city areas near the airport, whereas the previous studies on aircraft noise were conducted in the suburbs or towns located outside the city.

Sleep disturbance caused by aircraft noise is an important public health issue. In particular, the airport, on which this study was conducted, was located near the city with residents living in the area, and this might lead to more serious problems. The air services during the evening or nighttime also have a direct impact on the sleep pattern of the residents. For this reason, appropriate measures need to be considered.

## Conclusion

In conclusion, the prevalence of insomnia and daytime hypersomnia was higher in the residents, who are exposed to aircraft noise, as compared to the control group. This study was significant, as it was a large-scale epidemiological study. Further research needs to be conducted by using a direct measurement of the noise and objective sleep evaluation methods in order to clarify the cause-effect relationship.
